# Sporulation: A response to starvation in the fission yeast *Schizosaccharomyces pombe*

**DOI:** 10.1002/mbo3.1303

**Published:** 2022-06-21

**Authors:** Hokuto Ohtsuka, Kazuki Imada, Takafumi Shimasaki, Hirofumi Aiba

**Affiliations:** ^1^ Laboratory of Molecular Microbiology, Department of Basic Medicinal Sciences, Graduate School of Pharmaceutical Sciences Nagoya University Chikusa‐ku Nagoya Japan; ^2^ Department of Chemistry and Biochemistry National Institute of Technology (KOSEN), Suzuka College Suzuka Japan; ^3^ Department of Biology, Graduate School of Science Osaka City University Sumiyoshi‐ku Osaka Japan

**Keywords:** fission yeast, lifespan, *Schizosaccharomyces pombe*, sexual differentiation, spore, starvation

## Abstract

The fission yeast *Schizosaccharomyces pombe* employs two main strategies to adapt to the environment and survive when starved for nutrients. The strategies employ sporulation via sexual differentiation and extension of the chronological lifespan. When a cell is exposed to nutrient starvation in the presence of a cell of the opposite sex, the cells undergo fusion through conjugation and sporulation through meiosis. *S. pombe* spores are highly resistant to diverse stresses and may survive for a very long time. In this minireview, among the various sexual differentiation processes induced by starvation, we focused on and summarized the findings of the molecular mechanisms of spore formation in fission yeast. Furthermore, comparative measurements of the chronological lifespan of stationary phase cells and G_0_ cells and the survival period of spore cells revealed that the spore cells survived for a long period, indicating the presence of an effective mechanism for survival. Currently, many molecules involved in sporulation and their functions are being discovered; however, our understanding of these is not complete. Further understanding of spores may not only deepen our comprehension of sexual differentiation but may also provide hints for sustaining life.

## Graphical abstract

The fission yeast, *Schizosaccharomyces pombe*, employs two main strategies to adapt to the environment and survive when starved for nutrients: chronological lifespan extension and sporulation. In this minireview, we summarize several findings on spore formation in *S. pombe*. As observed, comparative measurements of the chronological lifespan of the stationary phase and the survival period of spore cells revealed that the latter achieved an extremely long survival period.

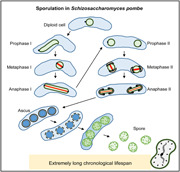

## 1 INTRODUCTION

### 1.1 The chronological lifespan (CLS) and sporulation of the fission yeast *Schizosaccharomyces pombe* in response to starvation

During starvation, *S. pombe* cells undergo sexual differentiation that leads to sporulation if appropriate conditions, that is, G_1_ arrest and the presence of opposite sex, are met, otherwise, cells often enter the stationary phase, in which the cell cycle is arrested at G_0_ with a relatively long CLS (Ohtsuka et al., 2021c; Otsubo & Yamamoto, 2012; Su et al., 1996; Tsuyuzaki et al., 2021). CLS is defined as the duration of survival of nondividing cells. In yeast, the survival rate after entry into the stationary phase is measured as the CLS (Kurauchi et al., 2017; Legon & Rallis, 2022; Lin & Austriaco, 2014; Longo et al., 2012; Matsui et al., 2021; Roux et al., 2010). At least 100 genes, 30 drugs, and approximately 10 nutritional conditions have extended the CLS in *S. pombe* (Emami & Ueno, 2021; Fujita et al., 2007; Hibi et al., 2018; Imai et al., 2020; Naito et al., 2014; Ohtsuka et al., 2021c; Romila et al., 2021; Su et al., 1996; Takuma et al., 2013). Therefore, a comprehensive understanding of CLS requires a detailed review of the biology and molecular mechanisms involved in the lifecycle of *S. pombe*. Although certain genes involved in sexual differentiation, such as Spk1 and Ste11, also affect CLS, numerous other factors that mediate the regulation of the CLS, such as the *ecl* family genes, a target of rapamycin complex 1 (TORC1), protein kinase A (PKA), Sty1, mitogen‐activated protein kinase (MAPK), and the transcription factor Phx1, also contribute to regulating sexual differentiation (J. Y. Kim et al., 2012a; Ohtsuka et al., 2021b). It has also been reported that several types of nutrient starvation conditions induce sporulation and extend the CLS of *S. pombe*. Hence, sporulation and CLS extension are considered survival strategies that allow *S. pombe* to retain its genetic information, even in resource‐poor environments (Ohtsuka et al., 2021c).

In *S. pombe*, sporulation occurs through gametogenesis during its sexual differentiation response (Shimoda, 2004). Although *S. pombe* normally proliferates as a haploid, exposure to nutrient starvation, such as nitrogen depletion, terminates mitosis; and the cells become diploid through conjugation, leading to an irreversible meiotic program (Dangarh et al., 2020; Yamashita et al., 2017). Thus, during meiosis I, homologous chromosomes adhere and separate into two nuclei (Yamashita et al., 2017). Sexual differentiation in *S. pombe* proceeds through mating (Seike, 2019; Sieber et al., 2022; Yamamoto, 2010) and diploid formation, including horsetail movement, meiosis, and recombination (Nambiar et al., 2019; Wells et al., 2006; Yamashita et al., 2017) and leads to sporulation (Shimoda, 2004). This review focuses on the molecular mechanism of sporulation, which has not been reviewed for approximately 20 years.

### 1.2 Mechanism of spore formation by *S. pombe*

**Figure 1:**
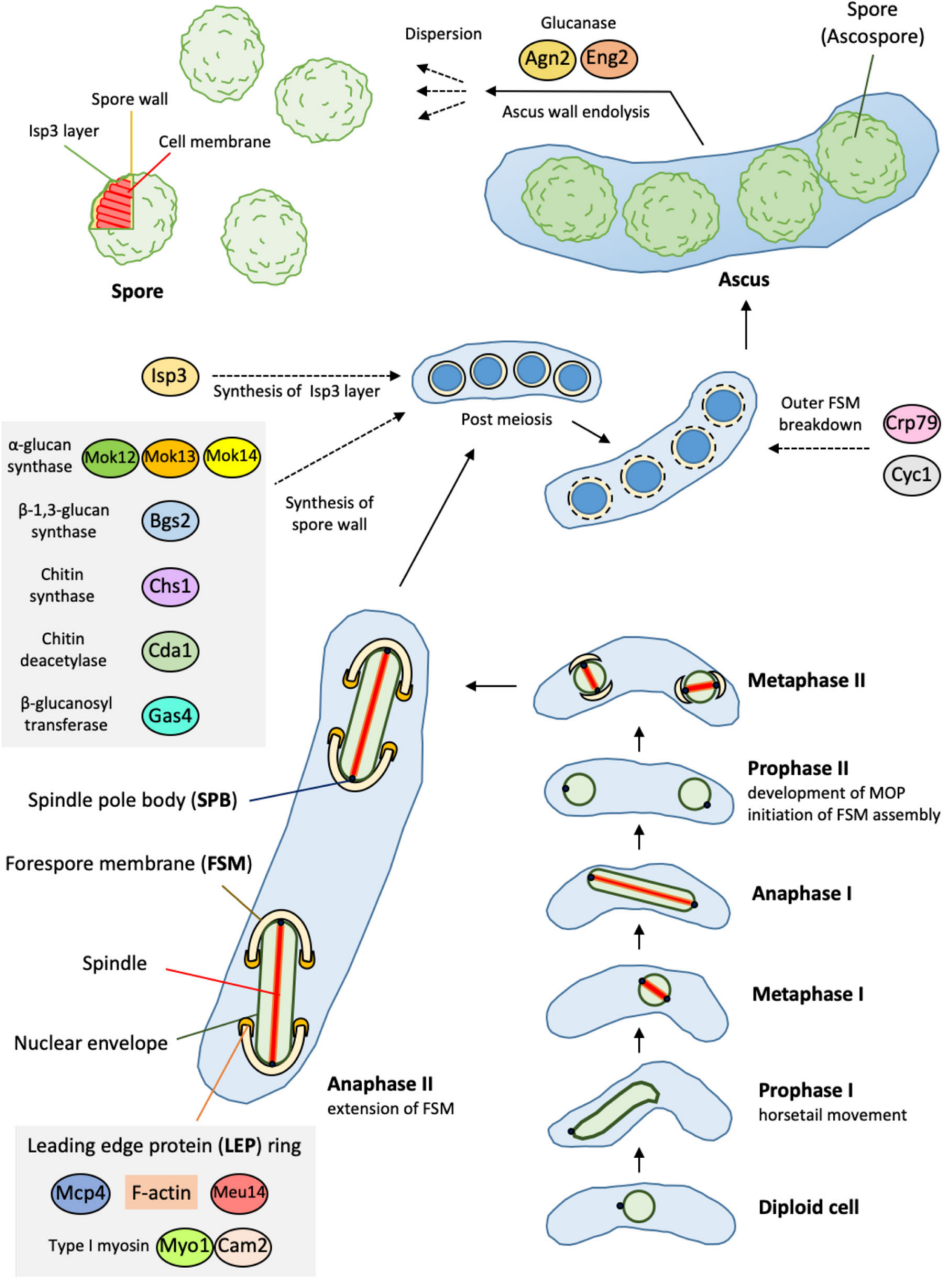
Sporulation by the fission yeast *Schizosaccharomyces pombe*. During starvation, diploid cells undergo meiosis for sporulation. Horsetail movement occurs in prophase I; meiotic outer plaques (MOPs) develop in prophase II and forespore membrane (FSM) assembly begins. In anaphase II, the FSMs created from the spindle pole bodies (SPBs) expand and wrap around the nuclei. At this time, the tip of the FSM is covered with leading‐edge proteins (LEPs). After meiosis, spore walls and isp3 layers are formed in the space within the FSM. The inner membrane of the FSM becomes the cell membrane of spores, and the outer membrane is degraded. The cell wall of the ascus is lysed by its enzyme, promoting the diffusion of spores. The details of each process are described in the text.

During the prophase of meiosis I, the elongated nucleus reciprocally moves (so‐called horsetail movement) (Itadani et al., 2010), while during meiosis II, chromosome segregation begins, and the two daughter nuclei divide (Yamashita et al., 2017; Yan & Balasubramanian, 2012) (Figure 1). Two spindle microtubules are assembled during prophase II to metaphase II (Takaine et al., 2014). During *S. pombe* sporulation, new daughter‐cell membranes are synthesized in the cytoplasm of the mother cell (Kashiwazaki et al., 2005). Two consecutive meiotic nuclear divisions produce four spherical prespores, each with one set of ploidy nuclei and organelles. During meiosis II, four double‐layered membranes (forespore membranes [FSMs]) are newly and simultaneously formed at each spindle pole body (SPB), which then encapsulate each nucleus (Imada & Nakamura, 2016; Niimi & Nakamura, 2018; Takaine et al., 2014). During anaphase II, the spindles elongate, the nuclei elongate and divide, and the FSMs elongate and wrap each nucleus (Takaine et al., 2014).

Unlike higher eukaryotes, numerous fungi, including *S. pombe*, undergo mitosis while retaining the nuclear envelope (designated "closed mitosis"), and proteins with a nuclear localization signal disperse into the cytosol during meiotic anaphase II (Arai et al., 2010; Asakawa et al., 2010). Further, meiosis II progresses without interpolar microtubules, and FSMs can generate a force to achieve SPB separation and nuclear division (Akera et al., 2012). Spore walls wrap the four haploid nuclei produced by meiosis to generate spores (Yamashita et al., 2017). For ascomycetes that do not produce conidia or chlamydospores, such as *Saccharomyces cerevisiae* and *S. pombe*, "spore" is synonymous with "ascospore," and we use "spore" hereafter. After sporulation, cells containing spores are called the ascus, a defining structure of ascomycetous fungi (Dekker et al., 2007). Thus, sporulation comprises the coordinated processes of meiosis and spore morphogenesis (Zhang et al., 2020). This review focuses on spore morphogenesis (Figure 1).

*S. pombe* forms spores through the steps as follows: (i) SPB modification, (ii) FSM assembly, and (iii) spore wall formation (Itadani et al., 2010). The proteinaceous SPB is the equivalent of the centrosome of animal cells (Nakamura‐Kubo et al., 2011). During interphase, the SPB resides in the cytoplasm close to the nuclear envelope in which it is embedded during meiosis (Nakamura‐Kubo et al., 2011). During prophase I, outer plaque begins forming on the cytoplasmic side of SPB (Nakamura et al., 2008).

During sporulation, mother cells synthesize the FSM, which subsequently serves as the spore's plasma membrane; and the SPB acts as a microtubule‐organizing center as well as a platform for FSM assembly (Nakamura‐Kubo et al., 2011; Shimoda, 2004) (Figure 1). When meiosis II commences, the single‐layer SPB embedded in the nuclear envelope doubles to form multilayered structures as follows: During prophase II, thick, multilayered, and disk‐type structures called meiotic outer plaques (MOPs) form on the cytoplasmic side of the SPB (called “SPB modification”) (Imada & Nakamura, 2016; Maeda et al., 2009; Nakamura‐Kubo et al., 2011; Takaine et al., 2014). During meiosis II, new double‐layered intracellular FSMs that subsequently cover the nucleus are formed (FSM assembly) as follows: After prophase II, FSMs commence assembly in the SPBs and expand through fusion with membrane vesicles derived from the endoplasmic reticulum (ER) and Golgi in synchrony with the formation of four prespores (Imada & Nakamura, 2016; Nakamura et al., 2008; Shimoda, 2004; Takaine et al., 2014; Wu et al., 2011; Zhang et al., 2020). During metaphase II, the FSM acquires a cup‐like structure (Takaine et al., 2014). Spore wall formation begins after FSM closure (Zhang et al., 2020), and the inner FSM layer becomes the plasma membrane of the spore, autolysis degrades the outer layer, and cell wall components are produced in the region between these two membranes (Itadani et al., 2010; Nakamura‐Kubo et al., 2011; Niimi & Nakamura, 2018; Zhang et al., 2020).

*S. pombe* spores are dormant cells resistant to diverse types of environmental stress (Tahara et al., 2020). It has been reported that fungi spores have high‐stress tolerance and are efficiently dispersed via gas or liquid (Brault et al., 2016). *S. cerevisiae* spores survive through the intestines of *Drosophila* (Coluccio et al., 2008). Furthermore, the spores of *S. pombe* are highly resistant to digestive enzymes and survive through the intestines of *Drosophila*, although their survival rate is less than half that of the spores of *S. cerevisiae*, suggesting that these spores contribute to dispersion by insect vectors (Coluccio et al., 2008; Tahara et al., 2020).

Outward protrusions cover *S. pombe* spores, the cell wall (spore wall) is thicker than that of a vegetative cell, and its constituents substantially differ (Tahara et al., 2020). The inner region of the spore wall contains β‐1,3‐glucan, and the outer region contains amylose‐like material (Medina‐Redondo de et al., 2008; Tougan et al., 2002). Approximately 46% and 38% of polysaccharides in the spore wall are α‐glucan and β‐glucan, respectively (Medina‐Redondo de et al., 2008). The cell wall composition of *S. pombe* includes galactomannan (9%–14%), α‐glucan (18%–28%), and β‐glucan (46%–54%) (Arellano et al., 2000; Matsuo et al., 2004), which accounts for the increased proportion of α‐glucan in the spore wall. Mutations in genes involved in the spore wall, including those that encode chitin synthase or chitin deacetylase, cause defects in spore wall formation, leading to a sensitivity of spores to stress, suggesting that the spore wall significantly contributes to stress tolerance (Arellano et al., 2000; Tahara et al., 2020; Zhang et al., 2020). Further, several deep and parallel invaginations are present on the plasma membrane surface of spores (Tahara et al., 2020) (Figure 1).

Aside from the importance of studies of the basic mechanisms and biological consequences of spore formation by yeasts, genomics research on yeast spores has contributed to a better understanding of the regulation of gene expression during cell differentiation (Shimoda, 2004). For example, studies of FSM assembly provide a model for de novo synthesis of membrane components in the cytoplasm (Ye et al., 2007).

## 2 MOLECULAR BASIS OF SPORULATION BY *S. POMBE*

Studies investigating nonessential gene‐deficient strains of *S. pombe* show that numerous genes are required for proper sporulation (Blyth et al., 2018; Ucisik‐Akkaya et al., 2014) (Table 1). Further, comparative analyses of fluorescent fusion proteins and proteins with known localization have greatly contributed to the understanding of the molecular mechanisms of sporulation (Imada & Nakamura, 2020; Shimoda, 2004; Yamashita et al., 2017). For example, the soluble N‐ethylmaleimide‐sensitive factor attachment protein receptor (SNARE) family member Psy1, Sad1 and Pcp1, and Cut11, are used to label the FSM, SPB, and nuclear envelope containing the SPB, respectively (Blyth et al., 2018; Maeda et al., 2009; Takaine et al., 2014; Yan et al., 2008).

**Table 1:** Factors involved in sporulation of the fission yeast *Schizosaccharomyces pombe*.

*S. pombe* protein	Mentioned part in the test	*S. cerevisiae* ortholog	*Homo sapiens* ortholog
Agn2	Spore wall formation	‐	‐
Avt3	Metabolism during sporulation	Avt3/4	SLC36A2/3/4
Bgs2	Spore wall formation	Gsc2, Fks1/3	‐
Cam1	SPB modification	Cmd1	CALM1/2/3, CALML3/4/5
Cam2	FSM assembly, leading‐edge proteins, and septins	‐	CALM1/2/3, CALML3/4/5
Cda1	Spore wall formation	Cda1/2	‐
Cdc7	FSM assembly and SIN	Cdc15	‐
Cdc11	FSM assembly and SIN	Nud1	CNTRL
Chs1	Spore wall formation	Chs1/2	‐
Crp79	Spore wall formation	‐	‐
Dma1	FSM assembly and SIN	Dma1/2	RNF8
Dms1	SPB modification	‐	‐
Eng2	Spore wall formation	Acf2, Dse4	‐
Fps1	FSM assembly and membrane trafficking	Erg20	FDPS
Gas4	Spore wall formation	Gas5	‐
Gdi1	FSM assembly and membrane trafficking	Gdi1	GDI1/2
Isp3	Spore wall formation	‐	‐
Mcp4	FSM assembly, leading‐edge proteins, and septins	‐	‐
Mei4	Metabolism during sporulation	Hcm1	‐
Meu10	Spore wall formation	Ecm33, Pst1 Sps2/22	‐
Meu14	FSM assembly, leading‐edge proteins, and septins	‐	‐
Mok12	Spore wall formation	‐	‐
Mok13	Spore wall formation	‐	‐
Mok14	Spore wall formation	‐	‐
Mug14	Metabolism during sporulation	‐	ADD1/2/3
Slk1	FSM assembly and SIN	Dbf2/20	STK38, STK38L
Myo1	FSM assembly, leading‐edge proteins, and septins	Myo3/5	MYO1E/1 F
Npg1	FSM assembly, leading‐edge proteins, and septins	Iqg1	IQGAP1/2
Pik3	FSM assembly, leading‐edge proteins, and septins	Vps34	PIK3C3
Psy1	FSM assembly and membrane trafficking	Sso1/2	STX1A/1B/2/4/11/19
Rgf2	Spore wall formation	Rom1/2	NET1, ARHGEF3
Sar1	FSM assembly and membrane trafficking	Sar1	SAR1A/1B
Section 2	FSM assembly and membrane trafficking	Section 2	RAB3IP RAB3IL1
Section 9	FSM assembly and membrane trafficking	Section 9, Spo20	SNAP23/25
Sid1	FSM assembly and SIN	Sid1	STK24/25/26
Sid2	FSM assembly and SIN	Dbf2/20	STK38, STK38L
Sid4	FSM assembly and SIN	Ady3, Cnm67	‐
Spg1	FSM assembly and SIN	Tem1	‐
Spn2	FSM assembly, leading‐edge proteins, and septins	Cdc10	SEPTIN9/12
Spn5	FSM assembly, leading‐edge proteins, and septins	‐	‐
Spn6	FSM assembly, leading‐edge proteins, and septins	Cdc12	‐
Spn7	FSM assembly, leading‐edge proteins, and septins	‐	‐
Spo2	SPB modification	‐	‐
Spo3	FSM assembly and membrane trafficking	‐	‐
Spo7	FSM assembly, leading‐edge proteins, and septins	‐	PSD, PSD2/3/4
Spo9	FSM assembly and membrane trafficking	Erg20	FDPS
Spo13	SPB modification	‐	‐
Spo14	FSM assembly and membrane trafficking	Section 12	PREB
Spo15	SPB modification	‐	‐
Spo20	FSM assembly and membrane trafficking	Ykl091c, Sec. 14	SEC. 14L1/2/3/4/5
Sst4	FSM assembly, leading‐edge proteins, and septins	Vps27	STAM, STAM2
Syb1	FSM assembly and membrane trafficking	Snc1/2	VAMP4
Vps5	FSM assembly, leading‐edge proteins, and septins	Vps5	SNX1/2
Vps17	FSM assembly, leading‐edge proteins, and septins	Vps17	SNX1/2
Vps29	FSM assembly, leading‐edge proteins, and septins	Vps29	VPS29
Vps33	FSM assembly, leading‐edge proteins, and septins	Vps33	VPS33A/33B
Ypt2	FSM assembly and membrane trafficking	Section 4	RAB8A/8B RAB10, RAB13
Ypt3	FSM assembly and membrane trafficking	Ypt31/32	RAB11A/11B
Ypt5	FSM assembly and membrane trafficking	Ypt52/53, Vps21	RAB5A/5B/5C
Ypt7	FSM assembly and membrane trafficking	Ypt7	RAB7A

Abbreviations: FSM, forespore membrane; SIN, septation initiation network; SPB, spindle pole body.

Sad1 mediates the insertion of the SPB into the nuclear envelope to facilitate the nucleation of mitotic spindle microtubules and facilitates centromere and telomere tethering during mitosis and meiosis (Varberg et al., 2020). The SPB component Cut11 mediates SPB insertion and acts on the nuclear envelope tethering of the nuclear pore complex (Varberg et al., 2020). Cut11 binds to the dynein light chain protein Dlc1, which localizes to the SPB throughout mitosis and meiosis and binds the meiosis‐specific SPB component Kms1 (Varberg et al., 2020). Dynein motors are required for horsetail movement during meiosis and to promote chromosome segregation during mitosis (Varberg et al., 2020).

### 2.1 Major events leading to sporulation

Sporulation has been reviewed below, with a focus on the major processes as follows: (1) SPB modification; (2) FSM assembly and membrane trafficking; (3) FSM assembly, LEPs, and septins; (4) FSM assembly and septation initiation network (SIN); (5) spore wall formation; (6) metabolism during sporulation; and (7) spore germination.

#### 2.1.1 SPB modification

**Figure 2:**
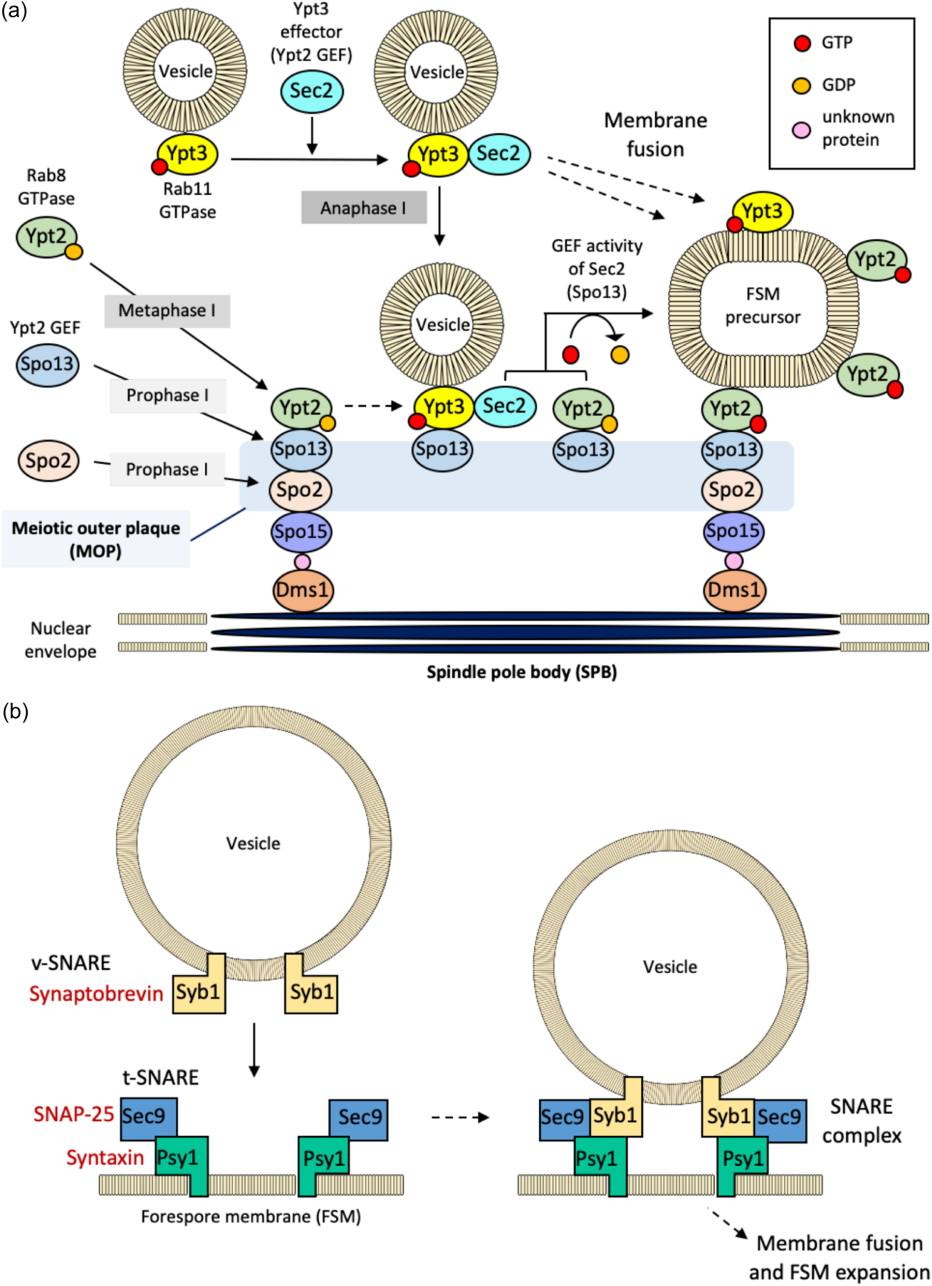
Molecular mechanisms of forespore membrane (FSM) formation in *Schizosaccharomyces ​​​​​​pombe*. (a) Initiation of FSM formation. During meiosis, I, meiotic outer plaques (MOPs) containing Spo2 and Spo13 are formed. Vesicles accumulate in MOPs through Rab family proteins, such as Ypt2 and Ypt3, and an FSM precursor is constructed. (b) The SNARE complex mediating FSM formation in fission yeast.

The MOP forms on the cytoplasmic side of the SPB that has invaded the nuclear envelope (Figure 2a). The MOP components Dms1, Cam1, and Spo15 prepare the foundation for subsequent cellular processes, including FSM assembly. Dms1, which binds to SPB components such as Cut11, localizes to the SPB and the nuclear envelope and likely controls spindle length and regulates the localization of Spo15 through physical interaction with Spo15 (Blyth et al., 2018; Niimi & Nakamura, 2018; Varberg et al., 2020). However, affinity capture‐mass spectrometry analysis indicates that Dms1 interacts with Spo15, although other pull‐down assays or yeast two‐hybrid assays do not detect a direct interaction, suggesting an indirect interaction between Dms1 and Spo15 (Niimi & Nakamura, 2018).

Vegetative growth and sporulation require calmodulin (Cam1), which localizes to the SPB after prophase I horsetail movement (Itadani et al., 2010). Therefore, cells harboring a Glu69 to Val mutation within the Ca^2+^ binding sites of Cam1 cause reduced sporulation (Itadani et al., 2010). SPB can be remodeled through a process in which certain SPB components transiently dissociate during the horsetail movement of prophase I. These components subsequently reassociate during meiosis I, mediated by a Polo‐kinase Plo1, which is then excluded from SPB in prophase I and moves to the kinetochore (Blyth et al., 2018; Ohta et al., 2012). Similar to SPB factors, such as Cut12 and Pcp1, the coiled‐coil protein Spo15 remains in the SPB during mitosis, dissociates from the SPB during prophase I horsetail movement, and returns to the SPB before the initiation of meiosis I (Ohta et al., 2012). Further, the localization of Spo15 to the SPB is Cam1‐dependent (Imada & Nakamura, 2016; Itadani et al., 2010).

Upon initiation of meiosis I, the sporulation‐specific protein, Spo2, and the Rab (Ras‐like in the brain) guanosine diphosphate (GDP)/guanosine triphosphate (GTP) exchange factor (GEF), Spo13, are recruited to the SPB in a Spo15‐dependent manner, which initiates MOP formation. Expression of *spo2* ^+^ and *spo13* ^+^ are also induced after meiosis I, favoring the binding of Spo2 to Spo15 on the surface of the SPB and the binding of Spo13 to Spo2 (Imada & Nakamura, 2016; Nakamura‐Kubo et al., 2011; Nakase et al., 2008; Shimoda, 2004). Therefore, deletion of SPB modification factors, such as Spo13 or Spo15, results in FSM assembly defects, indicating that SPB modification is required for subsequent FSM assembly (Ikemoto et al., 2000; Shimoda, 2004).

#### 2.1.2 FSM assembly and membrane trafficking

FSMs comprising double‐layered membranes are formed starting from modified SPBs; these are expanded by continuous membrane fusions via membrane trafficking. A Rab cascade regulates homeostasis and maintains a series of intracellular mechanisms through coordinated and dynamic intracellular membrane transport along the cytoskeletal pathway (H. Jin et al., 2021; Ohbayashi & Fukuda, 2013). Further, the Rab pathway mediates exocytosis and is involved in FSM formation (Imada & Nakamura, 2016) (Figure 2a). Rab GTPase mediates secretory vesicle transport, and Rab, which binds the transporting membrane vesicles, binds myosin V and moves along the actin filament to transport the vesicle to the target location (Y. Jin et al., 2011; Welz & Kerkhoff, 2019).

The Rabs in mammalian cells are called Ypts in yeast (Imada & Nakamura, 2016). A Rab cascade comprising the Rab family small GTPases, namely Ypt2 (human Rab8 ortholog) and Ypt3 (human Rab11 ortholog), and Section 2 (human Rabin8 or GRAB ortholog) that functions as an effector of Ypt3 and a GEF of Ypt2, is required for FSM formation in *S. pombe* (Horgan et al., 2013; Imada & Nakamura, 2016; Sato et al., 2007). Ypt2 and Ypt3 localize to the SPB in a Spo13‐dependent manner and control the initiation of the FSM assembly (Imada & Nakamura, 2016, 2020). Section 2 is partially recruited to the SPB, requiring Ypt3 (Imada & Nakamura, 2016).

Spo13 harbors a GEF domain required for FSM assembly and can act as a GEF for Ypt2 (Imada & Nakamura, 2016; Yang & Neiman, 2010). Spo13 also binds GTP‐ and GDP‐bound isoforms of Ypt3 (Imada & Nakamura, 2016). Ypt2 and Ypt3 are required for post‐Golgi membrane trafficking. Ypt3 localizes to cell tips and medial regions in vegetative cells and moves to the FSM during its assembly (Imada & Nakamura, 2016). In *Aspergillus nidulans*, a transport protein particle II (TRAPP‐II) acts as the GEF of the Rab11 ortholog (Pinar et al., 2015). In *S. pombe*, it has been observed that the TRAPP‐II complex and Ypt3 cooperate in vesicle transport (Wang et al., 2016). Additionally, in *S. pombe*, a GDP dissociation inhibitor (GDI) encoded by *gdi1* ^+^ is induced during meiosis and directly binds Ypt2 (Imada & Nakamura, 2016). GDIs here withdraw GDP‐bound Rab from the membrane after membrane trafficking, while retaining the prenylation (Imada & Nakamura, 2020; Ohbayashi & Fukuda, 2013). These interactions stably retain inactive Rab in the cytoplasm, allowing its reuse (Imada & Nakamura, 2020; Ohbayashi & Fukuda, 2013).

Rab family members bind the membrane via their geranylgeranylated posttranslationally modified C‐termini, which is essential for the proper localization of certain membrane proteins (Imada & Nakamura, 2016; Ye et al., 2007). In *S. pombe*, the complex comprising the farnesyl diphosphate synthase Fps1 and Spo9 exhibits geranylgeranyl diphosphate synthase activity and is involved in the secretory function and FSM assembly via protein prenylation (Ye et al., 2007). The geranylgeranyl diphosphate serves as a substrate for protein prenylation by geranylgeranyl transferase (Ye et al., 2007).

The interaction of SNAREs serves as a driving force for fusion between vesicles and membranes through the formation of a tetrapeptide helical bundle via the interaction of cytoplasmic domains (Holz & Zimmerberg, 2019). During FSM formation of *S. pombe*, FSM expansion is performed by fusing vesicles with the vesicle SNARE (v‐SNARE) Syb1, an ortholog of synaptobrevin, with the target SNARE (t‐SNARE) complex comprising the syntaxin ortholog Psy1 and the SNAP‐25 ortholog Section 9 (Maeda et al., 2009; Nakamura et al., 2005; Shimoda, 2004; Ucisik‐Akkaya et al., 2014; Yamaoka et al., 2013) (Figure 2b). Psy1 resides in the plasma membrane during vegetative growth and is recruited to the FSM precursor after meiosis I (Kashiwazaki et al., 2011; Maeda et al., 2009; Nakase et al., 2009; Shimoda, 2004). The *psy1* ^+^ gene was originally identified as a suppressor of spore defects in a *spo3* mutant (Maeda et al., 2009; Nakamura et al., 2001; Yamaoka et al., 2013). Spo3 is an FSM‐integrated meiosis‐specific coiled‐coil transmembrane protein that mediates membrane fusion during postmeiotic FSM expansion (Maeda et al., 2009; Nakamura et al., 2008). In *S. cerevisiae*, Section 4 (an ortholog of *S. pombe* Ypt2) functions in t‐SNARE Section 9 (an ortholog of *S. pombe* Section 9) (Brennwald et al., 1994; Y. Jin et al., 2011).

The Rab family GTPase Ypt5, an ortholog of Rab5, and vacuole‐localized protein Ypt7, an ortholog of Rab7 involved in endosome transport to vacuoles and homotypic vacuole fusion, are required for sporulation and FSM expansion (Kashiwazaki et al., 2005; Onishi et al., 2007, 2010; Tsukamoto et al., 2013). Ypt7 is activated by the GEF Vps39 (Kashiwazaki et al., 2005).

Spo14 and Spo20 mediate the supply of membrane vesicles to the FSM assembly and are required for proper FSM development (Maeda et al., 2009; Nakamura‐Kubo et al., 2003; Onishi et al., 2007; Shimoda, 2004). Spo14, an activator of the small GTP‐binding protein Sar1, which is essential for vesicle budding from ER, is required for the production of vesicles that mediate cargo transport between the ER and Golgi (Arai et al., 2010; Nakamura‐Kubo et al., 2003; Onishi et al., 2007). The phosphatidylinositol/phosphatidylcholine transfer protein Spo20 is essential for the formation of Golgi apparatus‐derived vesicles (Nakase et al., 2001; Onishi et al., 2007). Further, Spo20 alters its intracellular localization during meiosis and sporulation, is presented to cell poles during the mitotic cell cycle, moves to the nucleus during nutrient starvation, and to the newly formed FSM (Nakase et al., 2001; Takaine et al., 2014).

#### 2.1.3 FSM assembly, leading‐edge proteins (LEPs), and septins

Proper expansion of the FSM requires membrane‐bound complexes containing LEPs, which cover the leading edge of the FSM (Figure 1), and septins, a conserved family of GTP‐binding proteins, which contribute to FSM morphogenesis in parallel pathways (Onishi et al., 2010; Wu et al., 2011). The LEP Meu14, which harbors a coiled‐coil motif, first accumulates in the outer plaque of the SPB and then forms a ring structure at the leading edge of the FSM (Nakamura‐Kubo et al., 2011; Ohtaka et al., 2007; Shimoda, 2004). The LEP ring contains F‐actin and the meiotic coiled‐coil protein Mcp4. Further, a heavy chain of type‐I myosin Myo1 and the regulatory light chain Cam2, which is a calmodulin‐like protein induced by meiosis, colocalize with the actin structure in the region between Meu14 and Mcp4 (Itadani et al., 2006, 2010; Ohtaka et al., 2007; Yamashita et al., 2017; Yan & Balasubramanian, 2012; Yang et al., 2017).

During meiosis II, Meu14 is required for proper localization of Mcp4, which regulates the position of F‐actin during meiosis, and like Meu14, actin rings gather in the SPB early during meiosis II (Ohtaka et al., 2007; Yan & Balasubramanian, 2012). Another meiosis‐specific protein, Npg1, also contributes to FSM formation via proper development of leading edges and is involved in efficient spore formation and maintenance of viability. Npg1 genetically interacts with *meu14* ^+^ and *spo3* ^+^ from the assay using these double mutants, is transiently induced between meiosis I and meiosis II, and translocates from the nucleus to the FSM during its formation (Takaine et al., 2014).

Recruitment of Meu14 to the SPB and the leading edges of the FSM requires Spo7, which contributes to the initiation of FSM assembly and spore morphogenesis (Nakamura‐Kubo et al., 2011). Spo7 directly interacts with Meu14 (Nakamura‐Kubo et al., 2011; Takaine et al., 2014). While the factors required for SPB modification, such as Cam1, Spo2, Spo13, and Spo15, lack a lipid‐binding domain, Spo7 harbors a pleckstrin homology (PH) domain, which has an affinity for phosphatidylinositol 3‐phosphate (PI3P) (Nakamura‐Kubo et al., 2011; Onishi et al., 2003a). PI3P resides on the FSM (Onishi et al., 2003a). Spo7, which is transiently induced upon the initiation of SPB modification and FSM formation during meiosis II, and after meiosis I, also localizes to SPB independently of Cam1, Dms1, Spo2, Spo13, and Spo15 (Imada & Nakamura, 2016; Nakamura‐Kubo et al., 2011; Niimi & Nakamura, 2018; Takaine et al., 2014). Two‐hybrid analysis revealed that Spo7 physically interacts with other Spo7 molecules and binds Spo13, which is recruited to SPB before Spo7 (Nakamura‐Kubo et al., 2011) (Figure 2a).

The phosphatidylinositol 3‐kinase (PI3K) Pik3/Vps34, which catalyzes the production of PI3P, is required for proper FSM formation (Nakamura‐Kubo et al., 2011; Onishi et al., 2003a, 2007). Although PI3P is converted to phosphatidylinositol‐3, 5‐bisphosphate (PI3,5P2) by the phosphatidylinositol‐3‐phosphate‐5‐kinase Fab1/Ste12, PI3P but not PI3,5P2 may be required for sporulation, because a deletion mutant of *fab1* ^+^ sporulates (Onishi et al., 2003b). Localization of Meu14 in a deletion mutant of *pik3* ^+^ becomes abnormal, although Spo7 localizes to the SPB (Nakamura‐Kubo et al., 2011). Among targets of PI3P during sporulation, the sorting nexins Vps5 and Vps17 and the sorting receptor Sst4/Vps27 act downstream of Pik3 to regulate proper sporulation (Onishi et al., 2007, 2010). The retromer components Vps5, Vps17, and Vps29, which are involved in the retrograde transport from endosomes to the Golgi as well as the Section 1‐family protein Vps33 are required for proper FSM development (Ohtaka et al., 2008).

The *S. pombe* genome encodes the conserved GFP‐binding septins, Spn1–7 (Nakamura‐Kubo et al., 2011; Onishi et al., 2010). Spn1–4 assemble into a ring at the division site and form a hetero‐oligomeric complex, at the time of cytokinesis during vegetative growth (Onishi et al., 2010). The septin ring is important to properly target the endoglucanase to the division site (Onishi et al., 2010). During sexual conjugation, Spn1–4 assembles at the fusion site and are required for proper morphology (Onishi et al., 2010). In contrast, Spn2, Spn5, Spn6, and atypical Spn7, which are not included in the core subunits of the vegetative septin complex, are involved in sporulation and contribute to the accurate expansion of the FSM (Onishi et al., 2010). Spn2 and Spn5–7 are induced during sporulation, localize to the FSM (Nakamura‐Kubo et al., 2011; Onishi et al., 2010), and form a complex localized to a ring‐shaped structure along each FSM (Onishi et al., 2010). Spn2 and Spn7 bind to phosphatidylinositol 4‐phosphate (PI4P) which is abundant in the FSM in vitro, suggesting that these Spn isoforms bind the FSM via PI4P (Onishi et al., 2010).

#### 2.1.4 FSM assembly and SIN

*S. pombe* requires the SIN for spore formation (Krapp et al., 2006). The SIN controls the contraction of the actomyosin ring, the formation of new membranes, and septum division during mitosis. Further, the SIN is activated by meiosis II and contributes to FSM assembly during sporulation by regulating the closure rate of actin rings in LEP rings (Yan & Balasubramanian, 2012). The SIN is a GTPase‐regulated protein kinase cascade comprising the protein kinases Cdc7, Sid1, and Sid2, as well as the small GTPase Spg1 and the SIN scaffold proteins Cdc11 and Sid4 (W. Z. Li et al., 2010; Yan & Balasubramanian, 2012). These SIN factors are anchored to the SPB during meiosis via a SIN scaffold complex containing Cdc11 and Sid4 (W. Z. Li et al., 2010; Takaine et al., 2014; Yan & Balasubramanian, 2012).

During mitotic metaphase, the spindle‐checkpoint ubiquitin ligase Dma1 is recruited to the SPB, depending on Sid4 but not Cdc11, and negatively regulates mitotic exit and cytokinesis during late mitosis (W. Z. Li et al., 2010). During sporulation, Dma1 is transcriptionally induced at prophase I and regulates FSM assembly simultaneously with Meu14, septins, or the meiosis‐specific Sid2‐related kinase Slk1/Mug27/Ppk35 (Krapp et al., 2010; W. Z. Li et al., 2010; Takaine et al., 2014). Dma1 is recruited to the SPB during the early stage of FSM assembly, and SPB localization depends on Cdc11 and Sid4, but not other SIN proteins.

The roles of Slk1 and Sid2 during FSM expansion are redundant (Imada & Nakamura, 2020; Ohtaka et al., 2008). The Sid2 homolog Slk1, specifically expressed during meiosis, localizes to the SPB during prometaphase I in a SIN‐dependent manner and then translocates to the FSM during late anaphase II (Ohtaka et al., 2008; Takaine et al., 2014; Yan et al., 2008). Slk1 influences the development of the FSM by facilitating the recruitment of secretory apparatus components such as Psy1 in cooperation with Sid2 (W. Z. Li et al., 2010; Takaine et al., 2014; Yan et al., 2008). Although the SIN pathway kinase, Cdc7, is absent from SPBs during the horsetail stage and meiosis I but present during meiosis II, a mutation in Cdc7 leads to the formation of aberrant spores (Imada & Nakamura, 2020; Simanis, 2015).

#### 2.1.5 Spore wall formation

The spore wall of *S. pombe* comprises chitosan, mannan, and glucan, and the surface is covered with a proteinaceous fibrillar structure consisting mainly of Isp3 (Tahara et al., 2020). The Isp3 layer is interwoven and fibrillar (Tahara et al., 2020). The spore wall of *S. cerevisiae* is composed of β‐glucan, whereas that of *S. pombe* retains both α‐ and β‐glucans (Zhang et al., 2020). Spore wall synthesis requires the α‐glucan synthase subunits Mok12, Mok13, and Mok14; the β−1,3‐glucan synthase catalytic subunit Bgs2; chitin synthase Chs1; chitin deacetylase Cda1; and β‐glucanosyl transferase Gas4 (Encinar del Dedo et al., 2009; Martín et al., 2000; Matsuo et al., 2004, 2005; Medina‐Redondo de et al., 2008) (Figure 1).

Staining colonies with iodine have been established for easy identification of cell populations that are sporulating. During this process, the minor α‐1,4‐glucan polymer, synthesized by Mok14, reacts with iodine (Medina‐Redondo de et al., 2008; Yamashita et al., 2017). While α‐glucan synthase Ags1 is essential during vegetative growth, Mok12, Mok13, and Mok14, which localize to the spore envelope, are necessary for synthesizing α‐glucan in the spore wall (Medina‐Redondo de et al., 2008). The biosynthesis of the polysaccharide β‐1,3‐glucan in the spore wall is performed by the β‐1,3‐glucan synthase complex (García et al., 2009). The catalytic subunits of the β‐1,3‐glucan synthase complex are encoded by *bgs* family genes (*bgs1* ^+^, *bgs2* ^+^, *bgs3* ^+^, and *bgs4* ^+^) with GTPase Rho1 as its regulatory subunit (García et al., 2009; Liu et al., 2000). During sporulation, the synthesis of a linear β‐1,3‐glucan forming the fibrillar network is performed by the Bgs2 subunit of β‐1,3‐glucan synthase (García et al., 2009; Medina‐Redondo de et al., 2008). Bgs2 is induced during sporulation and localizes to the spore periphery (Medina‐Redondo de et al., 2008).

The Rho1 GEF, Rgf2 is involved in β‐glucan synthesis during sporulation and is required for spore wall maturation (García et al., 2009). During sporulation, the β‐1,3‐glucanosyltransferase Gas4 localizes to the spore periphery via the GPI anchor and is required for spore wall maturation and spore survival (Medina‐Redondo de et al., 2008). Further, the cell surface protein, Meu10, which is a homolog of both Sps2 and Sps22 that act on the organization of the β‐glucan layer of the spore wall in *S. cerevisiae*, is a component of the spore wall and may play an important role in the formation of the mature spore wall structure (Coluccio et al., 2004; Tougan et al., 2002). Deleting *meu10* ^+^ leads to abnormal localization of 1,3‐β‐glucan and delayed sporulation (Dudin et al., 2017; Tougan et al., 2002). The proper localization of Meu10 requires the expression of *apc14* ^+^/*omt1* ^+^ encoding the anaphase‐promoting complex subunit and of *omt2* ^+^, which is transcribed from the same genomic region as *apc14* ^+^ (Kakihara et al., 2003). Further, Mde10 functions as a Mei4‐dependent factor induced during spore formation, which is required for the ragged outer spore wall (Nakamura et al., 2004).

Breakdown of the outer FSM occurs during or after the formation of the Isp3 layer of the spore wall (Zhang et al., 2020). Mutations in *meu5* ^+^/*crp79* ^+^, which encodes an RNA‐binding protein, and *cyc1* ^+^, which encodes cytochrome *c*, do not disrupt the outer FSM. Evidence indicates that these factors contribute to the breakdown of the outer FSM (Zhang et al., 2020). Moreover, the level of Isp3 is significantly reduced in a deletion mutant of *meu5* ^+^ (Zhang et al., 2020).

After the spores have matured, the cell wall of the ascus undergoes an endolytic process mediated by the endo‐α‐1,3‐glucanase, Agn2, and the endo‐β−1,3‐glucanase, Eng2, for the release and dispersal of the spores, from the ascus into the environment (Dekker et al., 2007; Encinar del Dedo et al., 2009). Agn2 and Eng2 localize to the epiplasm, namely the cytoplasm of the ascus (Encinar del Dedo et al., 2009). Further, the defect in meiotic recombination activates the DNA synthesis checkpoint and suppresses the endolysis of the cell wall of the ascus (Guo & King, 2013).

#### 2.1.6 Metabolism during sporulation

When nitrogen starvation induces sporulation, cells acquire ATP mainly through glycolysis, and the levels of intracellular ATP decrease (Takaine et al., 2019). After sporulation, ATP levels are higher in the spores than in the epiplasm, and the ATP level in the ascus, excluding spores, is further reduced (Takaine et al., 2019). Moreover, during sporulation, the transcription factor Mei4 induces transcription of *ght6* ^+^ encoding a glucose/fructose transporter (Watanabe et al., 2001). The affinity of Ght6 for fructose is higher than that for glucose, leading to preferential uptake of fructose (Heiland et al., 2000). Since low glucose is a signal that induces sexual differentiation, including sporulation (Ohtsuka et al., 2015), cells may actively attempt to absorb carbon sources other than glucose that can be depleted in the environment.

Autophagy is a conserved process that delivers cytoplasmic components to the vacuole/lysosome (Nakatogawa, 2020; Xu & Du, 2022). Sporulation defects during starvation are exhibited by mutants with defective autophagy, suggesting that de novo protein synthesis is crucial for sporulation and that autophagy‐specific proteolysis contributes to the supply of nitrogen sources (Kohda et al., 2007; Mukaiyama et al., 2009, 2010). The sporulation defects of these autophagy mutants are recovered using exogenously added nutrients (Kohda et al., 2007). Further, some autophagy‐deficient cells complete sporulation, suggesting that such *S. pombe* cells store sufficient amounts of a nitrogen source to undergo sporulation, although the supply of a nitrogen source generated through autophagy contributes to sporulation (Mukaiyama et al., 2009). Moreover, the meiosis‐specific protein Mug14 influences the activity of the methionine salvage pathway during sporulation (Brault & Labbé, 2020), suggesting that this pathway contributes to the supply of methionine employed for de novo protein synthesis.

Under conditions of nutrient starvation during sporulation, vacuolar amino acids may contribute to protein synthesis. For example, a deletion mutant of Avt3, a vacuolar amino acid exporter involved in the export of basic and neutral amino acids from the vacuole to the cytosol, retains amino acids in the vacuole and decreases sporulation rate, possibly because amino acids cannot be used in the cytosol (Kawano‐Kawada et al., 2016).

#### 2.1.7 Spore germination

*S. pombe* spores germinate in response to glucose or sucrose but not fructose, maltose, or ribose (Johnke & Padilla, 1979; Kato et al., 2013). Germinated spores begin to exhibit polar growth and become markedly elliptical and elongated (outgrowth) (Johnke & Padilla, 1979). When spores germinate, the cell surface changes from a smooth state to a dendritic fibrous structure characteristic of vegetative cells (Tahara et al., 2020). Modulation of the expression of one of three copies of the histone H3 gene serves as a crucial trigger for germinating spores (Tsuyuzaki et al., 2020). Further, proper spore germination requires ferrichrome biosynthesis, a siderophore transporter Str1, cell surface Cu^2+^ transporters, superoxide dismutase 1, and the catalytic subunit of the β−1,3‐glucan synthase complex, including Bgs1, Bgs3, and Bgs4 (Cortés et al., 2005; Plante & Labbé, 2019; Plante et al., 2017).

During germination of *S. pombe* spores, trehalose is consumed mainly by the neutral trehalase Ntp1. In *ntp1*‐deficient cells, the initiation of germination is delayed, outgrowth is reduced, and the rate of intracellular trehalose reduction is decreased (Beltran et al., 2000). Ntp1 is posttranslationally activated upon an increase in the level of cAMP (Beltran et al., 2000). *S. pombe* expresses two trehalases, neutral trehalase and acid trehalase (Beltran et al., 2000). The aryl β‐d‐glucoside phloridzin, which inhibits sporulation‐specific acid trehalase, blocks germination in *ntp1*‐deficient cells, suggesting that these trehalases and trehalose utilization are required for the germination of *S. pombe* spores (Beltran et al., 2000). Trehalose 6‐phosphate synthetase, encoded by *tps1* ^+^, which converts glucose 6‐phosphate to trehalose 6‐phosphate, contributes to germination (Blázquez et al., 1994; Tsuyuzaki et al., 2021).

Mechanical stress caused by spore growth ruptures the spore wall, and the rupture determines the polarity of subsequent growth and development (Bonazzi et al., 2014). Analogous to vegetative cells, the growth of spores is powered by internal turgor (Bonazzi et al., 2014). Although the plasma membrane of a spore forms a highly folded structure (Tahara et al., 2020), this formation may aid rapid growth by omitting de novo plasma membrane synthesis during spore growth.

### 2.2 Sporulation‐specific signal transduction pathways in *S. pombe*

Signal transduction pathways that induce sporulation are as follows: (i) starvation signals and *ste11* ^+^ induction, (ii) response to mating pheromones, and (iii) stabilization of meiosis‐specific transcripts. These events are discussed in detail in this section.

#### 2.2.1 Starvation signals and ste11^+^ induction

The transcription factors Atf21, Atf31, Mei4, Rep1, and Ste11 are involved in the control of sexual differentiation (Mata et al., 2007; Morita et al., 2011). Among them, Ste11 is the first key transcription factor regulating sexual differentiation (Yamamoto, 1996). Ste11 is induced in response to nutrient starvation, after which it translocates from the cytoplasm to the nucleus, to induce the transcription of genes, such as *matP* ^+^, *matM* ^+^, *mei2* ^+^, and *sme2* ^+^, which are required for sexual differentiation (Mata & Bähler, 2006; Otsubo & Yamamoto, 2012; Qin et al., 2003; Sugimoto et al., 1991).

**Figure 3:**
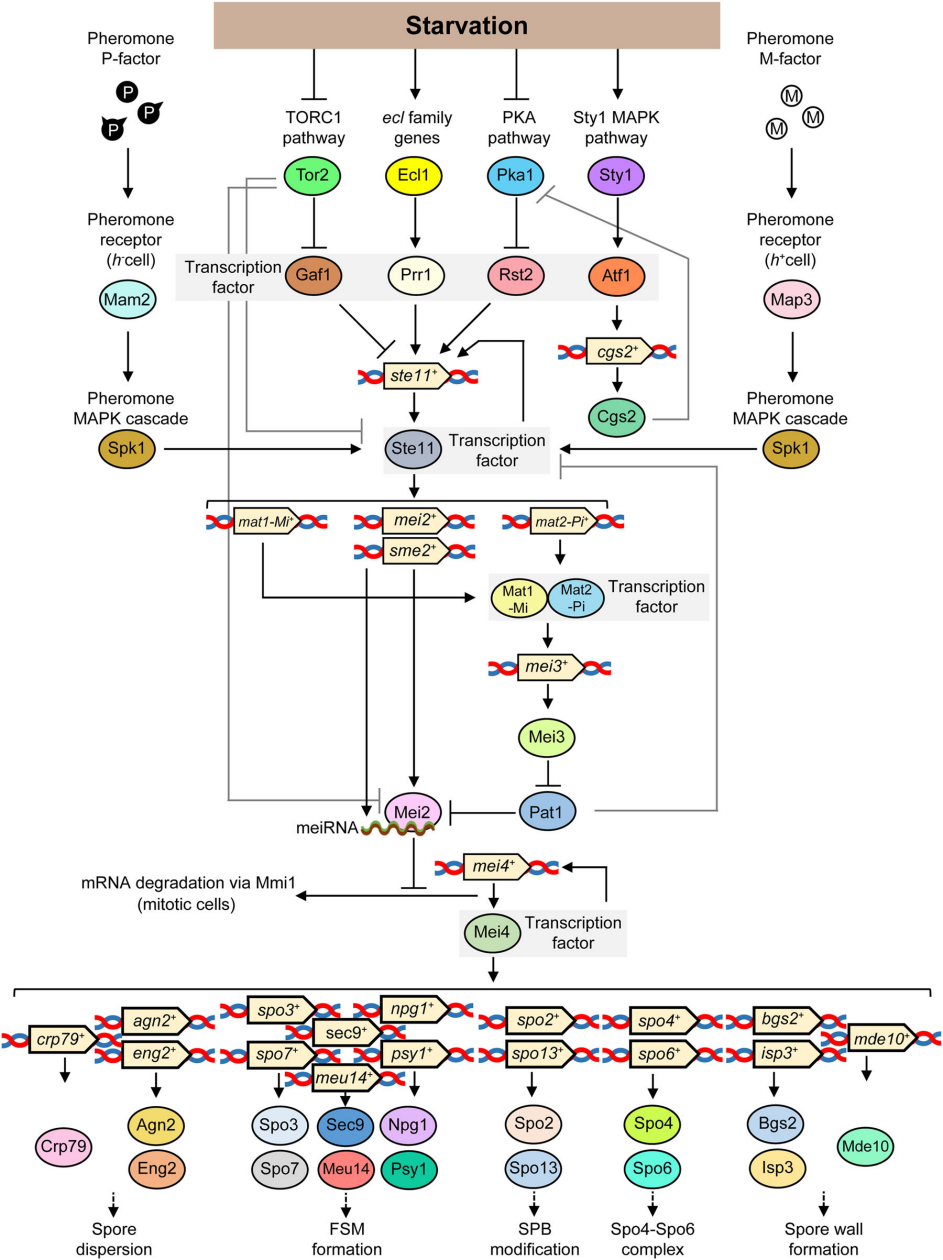
Signal transduction pathway required to induce sporulation in *Schizosaccharomyces pombe*. The details of each process are described in the text.

Various types of nutrient starvation induce the expression of *ste11* ^+^ through the signaling pathways as follows: TORC1, PKA‐Sty1 pathways, as well as by the *ecl* family genes (Gupta et al., 2011; L. Kim et al., 2012b; Ohtsuka & Aiba, 2017; Otsubo et al., 2017) (Figure 3). Among these signaling pathways, the expression of *ste11* ^+^ is positively regulated by the zinc finger transcription factor Rst2, which engages the PKA pathway, and the transcription factor Prr1, which acts downstream of *ecl* family genes, and is negatively regulated by the GATA family zinc finger transcription factor Gaf1, which engages the TORC1 pathway (Higuchi et al., 2002; L. Kim et al., 2012b; Ohmiya et al., 2000; Ohtsuka et al., 2012; Rodríguez‐López et al., 2020).

Ste11 binds upstream of *ste11* ^+^ to autoregulate its transcription (L. Kim et al., 2012b; Kunitomo et al., 2000). Although the Sty1 MAPK pathway is involved in the transcriptional regulation of *ste11* ^+^ (Kanoh et al., 1996; Shiozaki & Russell, 1996), the induction of *ste11* ^+^ by this pathway may represent an indirect process mediated via the PKA pathway. The transcription factor Atf1, which is activated by the Sty1 MAPK pathway, induces the transcription of phosphodiesterase Cgs2, a negative regulator of the PKA pathway (Sánchez‐Mir et al., 2020).

PKA targeted by cyclic AMP (cAMP) mediates the growth and sexual differentiation of *S. pombe* (Valbuena & Moreno, 2010). In the presence of sufficient nutrient levels, cAMP generated from ATP via adenylyl cyclase (Cyr1) binds to the regulatory subunit of PKA (Cgs1) and releases the catalytic subunit of PKA (Pka1), upon which Pka1 translocates from the cytosol to the nucleus (McInnis et al., 2010; Paul et al., 2009). Pka1 negatively regulates the transcriptional activity of Rst2, and Pka1 phosphorylates Rst2 to promote its translocation from the nucleus to the cytoplasm (Goldar et al., 2005; Higuchi et al., 2002). During nutrient starvation of *S. pombe*, Rst2 induces the transcription of *ste11* ^+^ by binding its upstream stress‐starvation response element (STREP) motif's consensus sequence CCCCTC (Higuchi et al., 2002; Otsubo & Yamamoto, 2012).

CLS is regulated by *ecl1* family genes in *S. pombe* and *S. cerevisiae* (Ohtsuka & Aiba, 2017). The Zn‐binding protein encoded by *ecl1* ^+^ (80 amino acid residues) is induced by various types of environmental stress, such as starvation (oxidative stress or nutrient starvation of nitrogen, amino acid, sulfur, or magnesium) (Miwa et al., 2011; Ohtsuka et al., 2019, 2021a, 2022b; Shimasaki et al., 2014, 2017). There are three *ecl* family genes, including *ecl1* ^+^ in *S. pombe* and one in *S. cerevisiae*, all of which contribute to the regulation of the CLS (Azuma et al., 2012; Ohtsuka & Aiba, 2017).

In *S. pombe*, all *ecl* family genes induce *ste11* ^+^ in a Prr1‐dependent manner (Ohtsuka & Aiba, 2017; Ohtsuka et al., 2012). Prr1 binds upstream of *ste11* ^+^, where a heat shock element is present, to positively regulate *ste11* ^+^ expression (Ohmiya et al., 2000). Further, *ecl* family genes are required to induce sporulation during sulfur starvation and induce sexual differentiation during iron or zinc starvation (Ohtsuka & Aiba, 2017; Ohtsuka et al., 2017). Although sulfur starvation induces autophagy and sporulation, and extends CLS, similarly to nitrogen starvation, it leads to neither G_1_ arrest nor mating (Corral‐Ramos et al., 2021; Ohtsuka et al., 2017, 2021d; Shimasaki et al., 2020). Recently, it was reported that *ecl* family genes inhibit TORC1 activity (Ohtsuka et al., 2022a).

In *S. pombe* cultured in the presence of sufficient nutrient levels, TORC1 promotes vegetative growth and suppresses sexual differentiation (Otsubo et al., 2018). In contrast, the target of rapamycin complex 2 (TORC2) is required for sexual differentiation (Otsubo et al., 2020). TORC1 promotes the phosphorylation of Gaf1 by suppressing PP2A‐like phosphatase Ppe1, leading to the cytosolic localization of Gaf1 (Otsubo et al., 2017). Gaf1 directly binds to the GATA motif upstream of *ste11* ^+^ to negatively regulate expression (L. Kim et al., 2012b). Further, TORC1 may directly suppress Ste11 activity via its phosphorylation, because the TORC1 kinase (Tor2) phosphorylates Ste11‐Thr88 and Ste11‐Thr82 in vitro (Otsubo et al., 2017). TORC1 directly phosphorylates and suppresses Mei2, which is essential for sexual differentiation (Otsubo et al., 2014). Moreover, overexpression of the transcription factor Fhl1 suppresses the hypersporulation phenotype of a *tor2* mutant, suggesting that Fhl1 may regulate the activities of pathways that control sexual differentiation downstream of the TORC1 pathway (Pataki et al., 2017).

*S. pombe* arrests the cell cycle at G_1_ and mates in response to nutrient starvation (Hayles & Nurse, 2018). Thus, mating only occurs during G_1_, in which cyclin‐dependent kinase (CDK) Cdc2 activity is low (Harigaya & Yamamoto, 2007), suggesting the regulation of mating by the cell cycle via CDK activity. Evidence indicates that Ste11 controls mating through two mechanisms as follows: (1) direct phosphorylation of Ste11 by Cdc2, which suppresses the DNA‐binding activity of Ste11 (Kjaerulff et al., 2007; Shimada et al., 2008), and (2) suppression of *ste11* ^+^ expression via the phosphorylation of the forkhead transcription factor Fkh2 by Cdc2 (Shimada et al., 2008). Although Fkh2 binds the FREAC‐like element of *spo6* (FLEX) sequence upstream of *ste11* ^+^ and contributes to the induction, Cdc2 suppresses the activity of Fkh2 via the phosphorylation of Fkh2‐T314 and Fkh2‐S462 (Shimada et al., 2008).

Expression of *ste11* ^+^ is also negatively regulated by the noncoding RNA, *rse1* (repressor of *ste11* expression), located upstream of *ste11* ^+^ (Fauquenoy et al., 2018; Yague‐Sanz et al., 2020); *rse1* recruits a protein complex that promotes deacetylation at the *ste11* ^+^ promoter site, possibly influencing the elongation rate of RNA polymerase II (Fauquenoy et al., 2018; Yague‐Sanz et al., 2020). Moreover, CDK Lsk1 regulates *ste11* ^+^ expression through the phosphorylation of RNA polymerase II (Coudreuse et al., 2010).

#### 2.2.2 Response to mating pheromones

Sex in S. *pombe* is a heterotypic event between a P‐cell and an M‐cell of different mating types, which involves the expression of numerous genes, including those encoding secretory pheromones and pheromone receptors (Vještica et al., 2016). The mating pheromones then induce sexual agglutination to form mating projections called "shmoo," in both M‐ and P‐cells (Seik et al., 2019). P‐cells produce 23‐mer peptide pheromones called P‐factors, which are recognized by the G protein‐coupled receptor (GPCR) Mam2 on the surface of M‐cells (Seike, 2019).

In contrast, M‐cells produce a farnesylated 9‐mer peptide pheromone called M‐factor, recognized by the GPCR Map3 on the surface of P‐cells (Seike, 2019; Seike et al., 2012). Precursors of P‐factors encoded by *map2* ^+^ are processed into three peptides to generate mature P‐factors (Seike, 2019). In standard laboratory strains, these pheromone‐coding sequences slightly differ, such that the mature P‐factor comprises three different peptides (Seike, 2019). In contrast, M‐factors precursors encoded by the functionally redundant genes *mfm1* ^+^, *mfm2* ^+^, and *mfm3* ^+^ mature through posttranslational processing, including farnesylation (Seike, 2019).

Pheromone‐receptor binding dissociates trimeric G protein subunits and activates the Spk1 MAPK cascade, which mediates pheromone signaling (Cansado et al., 2021; Seike, 2019). The Spk1 MAPK cascade comprises MAPKKK Byr2, MAPKK Byr1, and MAPK Spk1 (Gotoh et al., 1993; Kjaerulff et al., 2005). Spk1 activates Ste11 through phosphorylation of Ste11‐Thr305 and Ste‐Thr317 (Kjaerulff et al., 2005). Thus, the regulation of Ste11 is transcriptionally controlled by the starvation response as well as by the mating pheromone signal (L. Kim et al., 2012b; Kjaerulff et al., 2005).

Ras1, the only Ras GTPase homolog in fission yeast, is required for mating and the establishment of polarity during interphase (Khalili et al., 2018). Ras1 can activate the Byr2 component of the Spk1 cascade (Khalili et al., 2018; Otsubo & Yamamoto, 2012). In turn, GEF, Ste6, and the GTPase‐activating protein, Gap1, regulate the activity of Ras1 (Otsubo & Yamamoto, 2012; Papadaki et al., 2002). The expression of *ste6* ^+^ is induced by Ste11, indicating a positive‐feedback mechanism mediated by activation of the pheromone signaling cascade (Mata & Bähler, 2006; Otsubo & Yamamoto, 2012). Further, Byr2 is negatively regulated via localization mediated by the 14‐3‐3 proteins Rad24 and Rad25 (Ozoe et al., 2002). Moreover, the RNA‐binding protein Ndr1/Msa1, which is negatively regulated by the Spk1 cascade, coordinates with the ribosomal‐associated protein Cpc2 to regulate the levels of Ste11 (Oowatari et al., 2011). As described above, certain SPB factors containing Spo15 temporarily dissociate from the SPB during prophase I, and the Spk1 MAPK cascade contributes to SPB remodeling as well (Ohta et al., 2012).

#### 2.2.3 Stabilization of meiosis‐specific transcripts

The transition from mitosis to meiosis is mediated by Pat1 kinase, which acts as a brake for meiosis, and the RNA‐binding protein Mei2, which binds meiRNA expressed by *sme2*. During vegetative growth, Mei2 is phosphorylated by Pat1, loses its ability to bind to meiRNA, and undergoes ubiquitin‐dependent proteolysis (Goldar et al., 2005). In contrast, the dephosphorylation of Mei2 leads to the switch from the mitotic cell cycle to meiosis (Goldar et al., 2005; Kasama et al., 2006; Watanabe et al., 1997; Yamashita et al., 2017). Further, Pat1 suppresses the activity of Ste11 through the phosphorylation of Ste11‐Thr173 and Ste11‐Ser218 (Kjaerulff et al., 2005; McLeod et al., 2000). During the mitotic cell cycle, inappropriately expressed meiosis‐specific transcripts are recognized by Mmi1 and removed by nuclear exosomes (Yamashita et al., 2012). Mmi1 recognizes degradation signal sequences of transcripts, called "determinant of selective removal (DSR)" (Yamashita et al., 2012). Further, the Mlt1‐Red1 core (MTREC) complex acts as a bridge between nuclear exosomes and Mmi1 (Shah et al., 2014; Shichino et al., 2020). Thus, in mitotic cells, the system for selectively removing meiotic mRNA is regulated by the functions of DSR, Mmi1, and nuclear exosomes (Sugiyama & Sugioka‐Sugiyama, 2011). In contrast, when Pat1 is inactivated, Mei2 inhibits the DSR‐Mmi1 system by sequestering Mmi1 and secures stable expression of meiosis‐specific transcripts (Harigaya et al., 2006). A recent report indicates that TORC1 regulates MTREC via stabilization of its binding protein, Iss10, to control facultative heterochromatin and gene expression, including sexual differentiation (Wei et al., 2021).

Inactivation of Pat1 occurs through its binding to Mei3 (P. Li & McLeod, 1996; Yamashita et al., 2017), and *mei3* ^+^ is physiologically expressed only in *h* ^+^/*h* ^–^ diploid cells (Yamamoto, 1996). The products of *mat1‐Pi* ^+^ and *mat1‐Mi* ^+^ cooperatively induce the expression of *mei3* ^+^, which inhibits Pat1 kinase (Yamamoto, 2010). Concurrently, the bipartite Pi‐Mi transcriptional activator complex, which is formed after a fusion between the cytosolic M‐cell‐specific peptide Mi and the nuclear P‐cell‐specific homeobox protein Pi, is biased toward the P‐gamete side in the initial zygote and induces *mei3* ^+^ expression (Vještica et al., 2018). Subsequently, a zygotic transcription that drives meiosis initiates from the P‐parental genome (Vještica et al., 2018).

The *mei4* ^+^ transcript, which harbors DSRs regulated by Mei2, encodes the sporulation‐specific forkhead transcription factor Mei4, which binds a FLEX‐like element (Harigaya et al., 2006; Nakase et al., 2009; Takaine et al., 2014). Mei4 positively regulates transcription of the factors as follows: *spo4* ^+^ and *spo6* ^+^, which encode a component of the Spo4‐Spo6 kinase complex required for meiosis II and sporulation; *spo2* ^+^ and *spo13* ^+^, which are required for SPB modification; *meu14* ^+^, *npg1* ^+^, *psy1* ^+^, *Section 9* ^+^, *spn5* ^+^, *spn7* ^+^, *spo3* ^+^, and *spo7* ^+^, which are required for proper FSM formation; *apc14* ^+^, *bgs2* ^+^, *isp3* ^+^, and *mde10* ^+^, which are important for spore wall formation; *agn2* ^+^, *eng2* ^+^, and *meu5* ^+^/*crp79* ^+^, which are required for spore dispersal; and *mei4* ^+^ (Abe & Shimoda, 2000; Encinar del Dedo et al., 2009; Kakihara et al., 2003; Nakamura et al., 2000, 2002, 2004, 2005; WaNakamura‐Kubo et al., 2011; Nakase et al., 2008; Okuzaki et al., 2003; Takaine et al., 2014; Watanabe et al., 2001) (Figure 3). The gene *syb1* ^+^, encoding v‐SNARE, harbors upstream FLEX‐like elements, suggesting that induction during sporulation requires Mei4.

Mei4 regulates the expression of *wee1* ^+^, which regulates the cell cycle. When Mei4 binds the FLEX elements between *wee1* ^+^ and the adjacent gene *SPCC18B5.02c*, the expression of *SPCC18B5.02c* is induced and that of *wee1* ^+^ is simultaneously suppressed (Murakami‐Tonami et al., 2014). Inhibitory phosphorylation of CDK Cdc2 by the protein kinase, Wee1, occurs before meiosis I, followed by dephosphorylation of CDK Cdc2 by Cdc25 upon entry into meiosis I (MacKenzie & Lacefield, 2020). Evidence indicates that suppression of *wee1* ^+^ expression by Mei4 after meiosis contributes to the activation of CDK. Simultaneously, Cdc25 is induced during meiosis through a Mei4‐dependent mechanism (Murakami‐Tonami et al., 2007).

### 2.3 Other factors involved in sporulation

The *spo5* ^+^ transcript harbors DSRs and encodes the RNA‐binding protein Spo5, which regulates meiosis I; and deletion of Spo5 causes abnormal sporulation (Kasama et al., 2006; Yamashita, 2019). During prophase I, Spo5 forms a dot structure in the horsetail nucleus that colocalizes with the Mei2 dot (Kasama et al., 2006). The nuclear‐localized Mei2 dot structure of Mei2 and meiRNA is associated with the *sme2* locus of chromosome II (Yamashita, 2019). However, Spo5 acts on meiotic progression in a manner similar to, but independent of the Mei2/meiRNA complex (Kasama et al., 2006). Further, calcium transporting P‐type ATPase Cta4, which specifically localizes to the ER, is transcriptionally induced during meiosis and is required for FSM assembly (Yoshida et al., 2005). Further, Pom2, a dual‐specificity tyrosine phosphorylation‐regulated kinase (DYRK), is induced during meiosis, and when deleted, an abnormal ascus forms with defects in the FSM (Wu et al., 2011).

An increase in the activity of phospholipase D, which hydrolyzes phosphatidylcholine to generate phosphatidic acid and mediates lipid‐mediated signal transduction and membrane dynamics, occurs during conjugation and sporulation of *S. pombe* (Harkins et al., 2010). Further, evidence indicates that nitric oxide (NO) may regulate sporulation (Kig & Temizkan, 2009).

The addition of thiamine to culture media suppresses conjugation (McQuire & Young, 2006). Thi1, which regulates thiamine‐repressible *nmt1* ^+^, may positively regulate meiosis (McQuire & Young, 2006). The *nmt1* ^+^ promoter is widely used to drive gene expression in fission yeast. Although deletion of Thi1 causes defective sporulation and inhibits conjugation, Thi5, which controls the *nmt1* ^+^ promoter as well as Thi1, negatively regulates certain stages of meiosis (McQuire & Young, 2006).

## 3 SURVIVAL PERIOD OF *S. POMBE* SPORES

Nitrogen starvation, the optimum condition for extending CLS in *S. pombe*, arrests cells in the G_0_ phase to confer survival for approximately one month (Ohtsuka et al., 2017; Su et al., 1996). However, the dormancy of spores, including those of *S. pombe*, extends lifespan through an unknown mechanism (Maire et al., 2020). Furthermore, the duration of survival of *S. pombe* spores remains undetermined. Therefore, we approached this problem by determining the germination rates of spores harvested 96 or 57 months ago and stored in the dark at 4℃. As observed, 0.8% and 35% of these spores cultured in yeast extract complete medium germinated, respectively. Although cellular longevity can increase at low temperatures, spores not only enhance tolerance to stress but can also significantly extend the survival period. Furthermore, the long‐term survival of spores can be incomparably longer than the mitotic arrest period of vegetative cells in G_0_.

**Figure 4:**
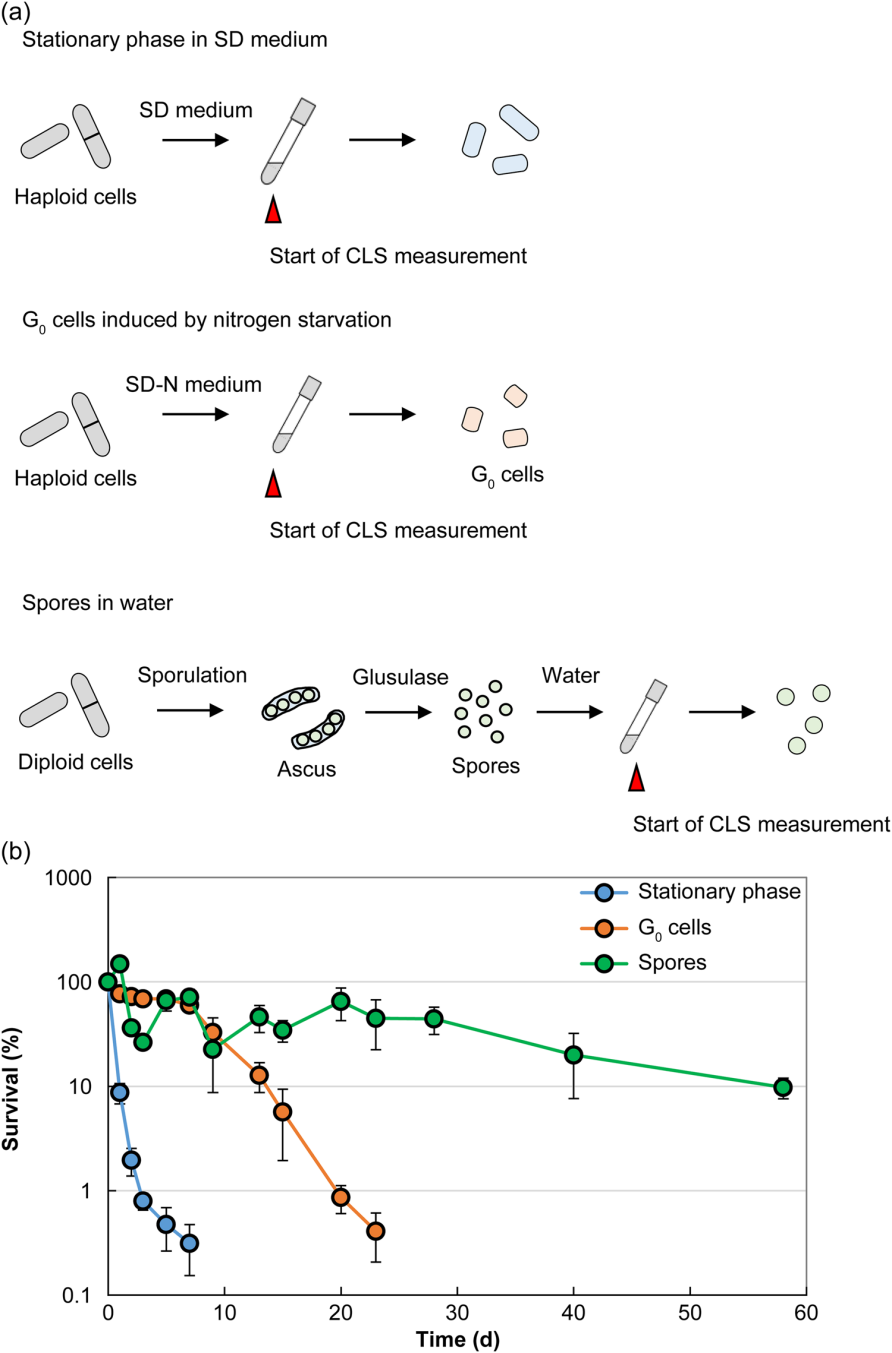
The survival period of *Schizosaccharomyces pombe* spores. (a) Overview of chronological lifespan (CLS) analysis. To determine the viability of cells in the stationary phase, the cells were grown in SD liquid medium. The determine the viability of G_0_ cells, the cells were grown in SD liquid medium until the OD of the medium was 0.5 and then transferred to SD liquid medium without nitrogen. For determining the viability of spore cells, they were cultured in water. All cell cultures were sampled in each growth phase and then plated onto yeast extract agar plates at a suitable dilution (Ohtsuka et al., 2021c). After incubation for several days at 30℃, the number of colonies derived from 1 ml of the culture suspension was counted. (b) The CLS of JY333 cells cultured in an SD medium (for stationary phase) and an SD medium without nitrogen (for G_0_ cells). Spores (from JY333 × HM3802 diploid) were cultured in water. Strains and media are described in Ohtsuka et al. (2017).

Therefore, we measured the survival period of *S. pombe* cells as follows: continuous normal culture, nitrogen starvation (the conditions that conferred the longest CLS), as well as the survival period of spores (Figure 4). During CLS under continuous culture, approximately 99% of cells die in approximately 1 week. In contrast, 99% of G_0_ cells starved of nitrogen die in approximately 1 month. In striking contrast, approximately 10% of these spores survived for 2 months. These findings indicate that *S. pombe* spores achieved the longest lifespan under these conditions.

Yeast cells, with an original CLS of approximately 1 week, survive for more than a month when subjected to adverse conditions such as nutrient starvation. However, despite the extensive studies reviewed here on the lifecycles of fission yeasts, the detailed mechanism that extends the survival period of spores is unknown. Although spores likely extend lifespan without utilizing external nutrients, lipid droplets, which are membrane monolayer organelles primarily comprised of the neutral lipids triacylglycerol and sterol esters, may contribute (Thiam et al., 2013; Yang et al., 2017). A study of the death of *S. cerevisiae* spores suggests that a chronological decrease in the molecules required for the transcriptional machinery reduces the expression levels of endogenous genes, which reduces the opportunities for germination, leading to death (Maire et al., 2020). More than 90% of *S. cerevisiae* spores in water at 30℃ die within approximately 2 months (Maire et al., 2020), which does not seem to be a major difference from the survival period of *S. pombe* spores (Figure 4), suggesting that the ability to extend the survival period by sporulation is comparable in these yeasts. Nevertheless, major questions, such as how spores maintain life, remain, despite the newly developed understanding of the factors that influence the survival of spores. The answers can potentially contribute a new perspective to future research on cellular lifespan.

## 4 CONCLUSION

Sporulation induces the expression of numerous genes not induced during vegetative growth, leading to highly specialized cellular processes, including the formation of new intracellular membrane structures, the FSMs. Numerous studies of sporulation identify the underlying molecular mechanisms. Yeast spores exhibit extremely strong stress tolerance compared with other cellular states. Further, analyses of the survival period of spores presented here show that spores experience a significantly longer survival period than other cellular states. The survival period of spores is significantly longer than the CLS of G_0_‐stationary phase cells subjected to nitrogen starvation, suggesting that sporulation involves unique mechanisms for maintaining life.

Nutrient‐starved yeast cells attempt to survive through the G_0_ entry phase or sporulation. These responses possibly influence the survival of this species. Furthermore, factors or processes involved in response to starvation, such as TORC1 and the PKA pathway, contribute to the control of lifespan from yeast to higher organisms (Folch et al., 2018; Hansen et al., 2018; Kapahi et al., 2017; Le Couteur et al., 2020; Ohtsuka et al., 2021c). Therefore, exploring the mechanism through which spores survive promises to contribute newer perspectives to the lifecycle of yeast as well as new applications and concepts that regulate longevity in higher organisms, including humans.

## AUTHOR CONTRIBUTIONS

**Hokuto Ohtsuka**: Conceptualization (lead); data curation (equal); funding acquisition (equal); writing—original draft (lead). **Kazuki Imada**: Data curation (equal); investigation (equal); writing—review and editing (equal). **Takafumi Shimasaki**: Investigation (equal); writing—review and editing (equal). **Hirofumi Aiba**: Funding acquisition (equal); writing—review and editing (equal).

## CONFLICT OF INTEREST

None declared.

## ETHICS STATEMENT

None required.

## Data Availability

All data are presented in this published article.
